# The Sufferings of the Biblical Job as an Icon of Postmodernity: The ‘loneliness’ of God and the human being in a consumerist paradise

**DOI:** 10.1007/s10943-021-01323-5

**Published:** 2021-07-03

**Authors:** Józef Stala, Elżbieta Osewska, Krzysztof Bochenek

**Affiliations:** 1 The Faculty of Theology, Section in Tarnów, The Pontifical University of John Paul II in Krakow, Ul. Kanonicza 25, 31-002 Kraków, Poland; 2 Faculty of Humanities, University of Applied Sciences in Tarnow, Tarnów, Poland; 3 College of Humanities, University of Rzeszow, Rzeszow, Poland

**Keywords:** Suffering—Postmodern society, Secularization—Transcendence

## Abstract

This article explores ways in which the attitudes of the biblical Job may enrich postmodernist philosophy by addressing some of its inherent problems. The discussion focuses in particular on the biblical Book of Job that can serve as an example of confronting suffering as a dramatic implication of human life that denies the sense of happiness. In an attempt to suppress this fear, the postmodern human contests, in various ways, the truth of their ontic frailty and the fragility of their constructed “happiness”. The questions that the biblical Job posed to God with a distinct air of resentment and regret seem at first sight to be meaningless as they are thrown into the void of a terrifying Universe. The critique offered here comes out of a Christian philosophical and theological base which posits that belief in the sacrum, transcendence, God and the hope of eternal life are key elements in a meaning system that fosters mental health and human happiness. In the postmodern system of meaning, individuals may no longer question the existence of God for the sake of human freedom, nor seek evidence of God’s non-existence, but simply live as if God does not exist. From a Christian perspective, it appears that non-belief in a transcendent spiritual dimension can inline people in postmodern society to feel that they live in an atmosphere of existential anxiety. Similarly, a Christian critique would consider that it is the postmodernist view of fluidity in all aspects of human life that leads to uncertainty and suffering, a causal consequence that people may not advert to. In this way, confronted with many postmodern phenomena, they may unknowingly live in a world of illusion. The Christian critique would also see it is as necessary and important to address constructively the challenges raised by cultural postmodernity. For this reason, the article will reflect on the realism of human suffering, the forgetting and rejection of God, as well as transcendence.

## Introduction

In the traditional society each person had their place, position, and belonging, which freed them from many anxieties and problems. The human person could develop, rise, and achieve greatness, but he could also undergo degradation (Osewska, 2019). Postmodernity changed all that was stable (Stala & Vodičar, 2018). From a Christian perspective, the person in postmodern society, lost in multiplicity, changeability, and fluidity, and deprived of order and harmony, struggles with increasing anxiety that cannot be avoided (Glasberg, 2012). “Standing before the abyss, the human looks into the deep, and what he sees at the bottom of the abyss is the tragic structure of existence. What becomes clear to him is that human existence is ultimately and most profoundly passion—that it is the nature of the human being to be a sufferer, a *homo patiens*.” (Frankl, 1971) Articulated so unambiguously by Victor Frankl, the idea of a close correlation between suffering and humanity was not and is not universally accepted. This dramatic vision of the human nature appears too pessimistic, far from the ideas that most people would like to exhibit in this regard. The drama of the thesis put forward by V. Frankl, who realistically considers suffering to be synonymous with the human, does not seem to be a problem of rationality or religiosity, but rather a problem of emotionality, which compels humans to seek “easy” happiness.

Many of the postmodernist philosophers offer ‘optimistic’ theses relating to the status of humanity; especially, those presented in a heavily trivialized version add little to the discussion of the meaning of human life. However, the question about the essence of humanity is thoroughly existential considerations, far exceeding the horizon of theoretical considerations. The perspective of understanding nature and humanity, even if completely unreflective, determines life decisions, hopes, and plans. Occasionally, the question about the real status of human nature appears with such an insistent brutality that one does not have to look for an answer anymore. It stops being an element of theoretical discussion, and becomes part of a life drama whose destructive power grows proportionately to the hypocrisy of the essence of humanity (Roy, 1991).

The present fluidity of all aspects of the modern human's life leads to uncertainty and suffering, which the person does not even notice. The contemporary human being, confronted with a multitude of postmodern phenomena, lives in a world of illusion, rejecting the truth about himself and leading to the objectification of himself (Helminiak, 2020). However, it is necessary to face the postmodern challenges and, for this reason, this article will reflect on the realism of human suffering and its undermining, the forgetting and rejection of God, as well as refer to Transcendence.

## The Dramatic Reality of Suffering

The biblical Book of Job serves as an excellent example of confronting suffering as a dramatic implication of humanity that denies the sense of happiness. The perspicacity of its analysis invariably inspires awe in people who ask about the misfortunes that afflict them (Roxberg and Brunt and Rask et al. 2013). The life of the biblical Job and the dilemmas that tormented him are the story of human life, repeated in billions of copies. Regardless of the era, the same unfortunate events occur over and over again and cannot be grasped by reason or accepted on the level of will, emotions, feelings, and, finally, faith (Johnson, 2005). From the Christian philosophical perspective, the contemporary Job feels unhappy, suffers, and finally dies from malnutrition, innumerable diseases, incomprehension, lack of love, hatred, but also egoism, lack of any axiology, or lack of deeper reflection on himself, the world, and God. When he leads a happy life, even if only seemingly, he probably doesn't think often about the meaning of suffering and doesn't categorize happiness and unhappiness. Nor does he argue with God about why he is happy. He turns to Him only when he is afflicted with helplessness in the face of a series of misfortunes that devastate him. Increasingly more often, especially in Western civilization, “sterilized” from God and the devil, transcendence and spirituality, the postmodern Job faces his pain of existence alone. Just as “lonely” God seems to be in the postmodern world, as he does not find many reliable witnesses today.

Christian philosophers highlight that the postmodern human finds it increasingly difficult to identify with the biblical Job, the “victim” of a kind of “wager” between God and Satan, questioning the purity of his intentions with regard to suffering. The biblical figure, however, consistently believed that he could not plead his innocence and sinlessness, while suffering such striking misfortunes (Kwon, 2020). Even if these dilemmas are distant to the postmodern Job, who overestimates his ontic and moral status, this story delivers the synthetic truth about the fate of every human being, without exception. In addition, the dramas of billions of Jobs alive today are compounded by the phenomena that constitute postmodernity: secularization, the preference for a reductive anthropology, technological developments devoid of ethical context, and consumerism that questions any value of suffering. Constantly confronted with the reality of suffering and unable to give it a true meaning, he often creates more or less sophisticated illusions of suffering, or exists in the sense of the absurdity of his own existence.

The eternal hopes of the human for a happy and eternal life in the consumerist paradise are invariably thwarted by the reality of suffering, old age, incurable illnesses, and above all, even if subjectively distant yet inevitable, death. Even if one tries to contest this, it is precisely these facts that constitute the dramatic dimension of humanity. The confrontation with various extreme situations is always a shock with which the human cannot cope, neither in terms of reason nor faith. In spite of the fact that suffering is the only unproblematic or, indeed, absolutely certain fact of his life, he always sees something profoundly inhuman and unnatural in it. And yet, as Arthur Schopenhauer states with brutal but suggestive frankness, “We are like lambs in a field, disporting themselves under the eye of the butcher, who chooses out first one and then another for his prey.” (Schopenhauer, 1995).

Even people who are the envy of others because they seem to lead happy lives must sooner or later realistically realize that, taken as a whole, they are a kind of mystification with a sad ending. Even if at some point in life someone thinks he or she is happy, there is unhappiness lurking somewhere nearby, from which there is no escape (Levinas, 1999). This is why, in spite of the growing comfort of life, postmodern Job is ruled by a paralysing fear of losing their fickle happiness. Probably even greater than in previous eras, in which the vast majority of people identified “happiness” with mere survival.

## The Postmodern Contestation of Suffering

A Christian critique of the postmodern situation offered in this article recognizes that in an attempt to suppress this fear, the postmodern human contests, in various ways, the truth of his ontic frailty, and the fragility of the “happiness” he constructs. This is not, of course, a tendency previously unknown. After all, existence in paradise, where no sad events interfere with joyful consumption, is the eternal objective of human's efforts. However, having completely different possibilities than his predecessors in this area, due to the democratization of life and the progress of technology and civilization, the postmodern human feels more entitled to dream such dreams. Christian philosophers and theologian notice that despite the fact that suffering constitutes the “world” of human existence, it is nowadays ‘censored’ and rejected as meaningless because it annihilates life in a consumerist paradise. The suffering, the old, the disabled, the handicapped, and the dying are increasingly isolated from a society that seeks to consume its goods peacefully and without any distractions. Those who are marked by suffering are incontestably “different” from “happy” consumers; thus, they invariably arouse fear of something difficult to accept. *Homo ludens*, trying to consume the goods offered on the outside without unnecessary hesitation, does not need to reflect on his Job-like status. Neither does he want to see around him “the unfortunates” who epitomize defeat and remind him of the fact that he is, in fact, very little different from them (Bajda, 2020).

According to Christian philosophers, many postmodern people having no possibility to fully overcome the drama of existence, if only through faith, or at least to somehow rationalize it and make it real, they try to convince themselves that the “pain of existence” does not concern them. A natural reaction to what does not fit into the illusion of a happy consumer is an attitude of mental and emotional escape from an “alien” reality. Preferring egoistic hedonism as a remedy for the pain of suffering, *homo consumens*, who does not want to be Job, tries to build a feeling of a permanent “carnival”. He is not even particularly original in this, as evidenced by Blaise Pascal's gloomy statement from the middle of the seventeenth century: “As men are not able to fight against death, misery, ignorance, they have taken it into their heads, in order to be happy, not to think of them at all.” (Pascal, 1989).

Immersed in consumption which, by its very nature, prefers pleasures of the flesh, the postmodern human ceases to feel hunger for what is spiritual, subtle, and unobtrusive. Contrary to what one might expect, spiritual hunger exceeds the desire for material things, even food, when one finds oneself in extreme circumstances. However, it can fade or even die out in a situation where one's sense of “happiness” seems to be undisturbed by anything. An evocative example of this is the story of Victor Frankl, a prisoner of a Nazi camp, who recalled how, in the dramatic circumstances of camp life, heated philosophical discussions arose, which he identified with a spiritual tendency for self-preservation (Frankl, 1971).

Christian philosophy reminds that the postmodern, trivialized popular culture is not meant to initiate this type of discussion; it avoids or eliminates topics which could “disturb” audiences, thus “helping” them to function. The reverie about suffering, which leads to rather painful conclusions, appears to be a commodity that may be of interest to a small number of customers and will not balance out financially, even if the optimistic messages seem absurd when confronted with the data of existence. The common consumer of culture, as strongly argued by Zygmunt Bauman, is nowadays being trained discretely, though consistently, to stop thinking about something he has no chance of obtaining anyway. Therefore, he ceases to desire eternal life properly, accepting that it is impossible to attain. By dismantling one by one, each floor of the elaborate building that the Church has erected over the centuries, modernity has also suppressed the fascination with the afterlife, focusing the human's attention on living only “here and now.” (Bauman, Tester, 2003) According to some postmodern experts, fears which torment the human in this area are to be replaced by “eschatological hopes”, based on scientific achievements, e.g. in the field of medicine. By building a happy order based on the previously unknown possibilities of science, suffering is not to be seen as the synthesis of life, but its opposite, the failure of medicine to “repair” the body. It is not uncommon for technology to no longer serve human, but to deepen dehumanization by "supporting" him in building a wall against the sad aspects of life. In a technicized world, which prefers empirically founded rationality, suffering loses its dimension of mystery and becomes a brutal fact, reminding us of the breakdown or disintegration of some device (Marcel, 1995). Suffering should, of course, be analysed as an objective fact, but there is however, a fundamental difference between analytical knowledge in this regard and an intimate, “personal”, existential experience (Foley, 1988). Even if the medical progress favours the deconstruction of suffering and death and is able to minimize pain, it is, as Z. Bauman insightfully notes, a life supervised from beginning to end, by garrisons left behind by a seemingly chased away enemy (Bauman, 2000). This is why postmodern Job, escaping from the status defined by this concept, does not cease to carry within him the drama expressed by his susceptibility to suffering. Existing in a consumerist, narcissistic society, he does not want to grow up, take responsibility, face what is difficult and, above all, confront his suffering and mortality.

While the biblical Job, convinced of his righteousness, is tormented by his inability to make sense of the misfortunes that befall him, the postmodern Job, who is focused on pleasure, wants only a life without suffering even if he knows that this is not possible. This avoidance of discomfort, especially when associated with suffering, coincides perfectly with the ideas of market and democratic reality. After all, their goal is nothing other than to reassure us that, as unique beings, we deserve “happiness”.

## Secularization

Although every human being is forced to identify with the drama of the biblical Job, unlike him, he is increasingly condemned to solitude in this aspect/area. The questions that the biblical Job posed to God with a distinct air of resentment and regret, as did billions of other people, seem to be meaningless as they are thrown into the void of a terrifying, soulless Universe. In postmodernity, one no longer questions the existence of God for the sake of human freedom, as Friedrich Nietzsche or Jean-Paul Sartre did, one no longer seeks evidence of his non-existence, but simply lives as if God does not exist. Preferring an inertial and unreflective atheism, the postmodern person tries to “believe” that he is not a pilgrim (*homo viator*), but an entity that sets itself the goal of its own life's journey. His “secular pilgrimage”, which rejects such traditional directions as the Promised Land, an encounter with God, or eternal existence, usually does not go anywhere, orienting itself towards immediate benefits (Życiński, 2001).

Rejecting his status as Job, the postmodern human wants no explanations from God; he expects only a life without suffering. Indeed, one who cries out and argues with God is the one who believes in Him, trusting ultimately that even though misfortune has befallen him, he is in His good hands. A Christian critique of the postmodern situation considers that problems arise where people today do not even fight with God, they do not question his existence argue about the justice of his decisions, and even if a large group of people do not openly contest the existence of God, then, in principle, they do not pay attention to Him. Many are trying to find a worthy place in the consumerist paradise, which is so real that they have actually forgotten about the vague promise of eternal happiness. Likewise, they have dismissed religious “oppressions” which do not fit into the consumerist reality: sin, penance, asceticism, humility. The dispute over transcendence seems to be coming to an end, and ‘the Kingdom come’ finally disappears on the horizon of the consumerist heaven. While the postmodern human recognizes, God as a fatal delusion (Dawkins, 2007), often even obsessively as Richard Dawkins does, he creates for himself various types of “idols” attributing salvific connotations to various forms of material prosperity.

In the secularizing postmodern world in which suffering is still present, many people have lost hope in the existence of Someone who can explain it in a meaningful way – the great, hidden providential Physician who makes sense of every event (Kołakowski, 1994). Particularly in economically developed countries, there is an attempt to believe in new forms of salvation, which derive and outcome of science: the development of technology, medicine or psychology (Kołakowski, 1999). Admittedly, as pointed out by Peter Atkins, science grew out of religion, relying on faith as it did, but when it “discarded its chrysalis to become its present butterfly, it took over the heath. There is no reason to suppose that science cannot deal with every aspect of existence.” (Atkins, 1995).

The progress made possible by science, the postmodern substitute for God, this “new incarnation” does little to explain the reality of suffering. In addition, the promises of “salvation” which is preferred today and which is frequently overlooked, also produce a system of prohibitions and precepts of an almost absolute character. The consequence of eliminating the realm of the sacred is pseudo-sacralization, a caricatured, dramatically banal, and even irrational imitation of eternal salvation. In the postmodern “temples” of consumption – the shopping malls – the human person no longer has to struggle with moral prohibitions or precepts, a sense of sin or remorse, but can “enjoy” life, here and now, unreflectively and without problems. In fact, the “only”, undeniably serious limitation of “religion” remains financial status. “Just as medieval society was balanced on God and the Devil, so ours is balanced on consumption and its denunciation. Though at least around the Devil heresies and black magic sects could organize. Our magic is white. No heresy is possible any longer in a state of affluence.” (Baudrillard, 2006) The lack of contention between faith and reason indeed favours neither reason nor faith. These two realities need each other. In the context of the struggle between reason and faith, it is easier for postmodern *homo patiens* to find space for a meaningful life. Confronted with suffering, he cannot abandon reason, nor can he unreflectively exclude God and faith.

## Transcendence

Sooner or later in life, every human being encounters extreme situations from which, unfortunately, there seems to be no escape. At such moments, marked by a sense of hopelessness, the question arises as to whether the limits of human nature are defined by its biological nature or whether there is a space of spirituality through which a person can transcend suffering. This way of transcendence, overcoming fears and ontic limitations, is determined by the axiological context of the existence of a person (Healy, 1995). For these reasons the Christianity attaches great importance to the human person's dignity based on the relationships with God. In the world of values, suffering acquires a connotation unknown in extra-human reality. By facing suffering and, in a sense, accepting it, the human person gets a chance to enter the path of development of his personality (Stala, 2012). There can be no question of the human's maturity as a person if he does not try to transcend his egoism through love, offering support to other suffering human beings. Since every person remains a ‘Job’, whether he/she wants to acknowledge it or not, it is evident that suffering has a social and communal dimension. Since the dawn of humanity, humans have tried to explain the drama of their misfortunes through faith and community with God.

Socrates, Francis of Assisi, or Gandhi did not perceive the evil that befell them as an ultimately unjust event, affecting someone despite his innocence or even high moral ground. Representing the highest moral and religious standards and, at the same time, evaluating realistically the status of being, they did not contest the meaning of suffering, nor did they intend to argue with God because of it. They were able to discover the profound meaning of suffering, because they opened themselves wide to their human manifold potentiality (John Paul II,+ 2001).

This was the attitude of Job, who rejected the theory of suffering as just punishment, which still has its adherents. He knew full well that misfortunes are distributed randomly, so they cannot be explained in terms of merit or rewards and punishments. He was a noble human all his life and God, whose ways are incomprehensible to us, knew this. Suffering is inexplicable precisely because it has nothing to do with justice, truth, or even the moral condition. It is absolutely individual and intimate, impossible to objectify and rationalize (Parker, 1997). The unhappy person is absolutely convinced that no one can understand the enormity of his pain, because no one experiences it on such a large scale. The experience of other people ceases to have any meaning here; it becomes illusory, inadequate, explaining nothing. The experience of real suffering, real of course in its subjective sense, leads the person beyond the safe and peaceful world of universal, objective explanations of himself and the world. According to Christian philosophical research, it is only when *homo consumens* collides with suffering, when he discovers in himself the Job he never wanted to become, that he becomes a human being conscious of himself, of his ontic status. Postmodern Job begins to understand himself when he realizes his limitations, especially the most important one, concerning the insignificance of his existence.

Christianity recognizes that the road to gaining this awareness is not easy, because the understanding of human today is so often dominated by the perspective of determinism, which narrows his perception to corporeality, psychology, or social relations, invoking important aspects of humanity, but not exhausting its essence. This overlooked element in the description of human nature is the spiritual sphere, i.e. the intentionality that characterizes the human being, the striving for meaning and values, particularly the absolute ones. An integral vision of human nature makes it possible to transcend the leading contemporary proposals that are antithetical to one another: nihilism, which completely devalues human, and humanism, which attributes an almost divine value to him/her. Immersed in the world of nature and society, and, at the same time, transcending these determinations through spirituality, the human can realize that he is a being in the middle; he is neither nothing nor everything (Frankl, 1971).

By courageously overcoming the fear of suffering, which is demanding, stripped of all sentimentality, meaning and beauty, *homo patiens* can maintain a nurtured openness to every experience, including that of suffering. He carries within himself, the capacity to go beyond himself, beyond his sense of unhappiness, of injustice, to discover humility, friendship, gratitude and, above all, the transcendent meaning of suffering. The condition for this discovery does not have to be religious faith, but it can be grounded on values, the idea of sacrifice and heroism. The human's life retains its value even in unbearable circumstances because it carries the capacity to creatively transform the negative aspects of life into something positive and constructive within him. This potentiality, however, can only be realized when he is able to make sense of his unhappiness, to fill it with intention, to transcend it, to suffer for something or for someone, to rise higher, upward, and forward, to stand “above” pains and misfortunes (Frankl, 1971). Although he may be unhappy and sad in life, he will not lose power over his existence and will not fall into despair implied by the absolutization of some relative value and the loss of the proper hierarchy of values.

The postmodern Job, like the biblical Job, will not find the meaning of his misfortunes through sophisticated reflection or through the revelations and instructions formulated by the various religions, but only in actions resulting from a deep faith. For this very reason, professing faith alone, even if accompanied by respect for the moral norms, is not yet a remedy for human suffering, nor does it offer a chance for its transcendence. For it is not a matter of a faith, but a strong faith. There can be no question of an automatic, problem-free remedy for the elimination of suffering.

## Conclusion

The critique offered in this article comes out of a Christian base which posits that belief in the sacred, transcendence, theism, and the hope of new, eternal life are key elements in a meaning system that fosters not only physical and mental health, but also human happiness. As the world becomes increasingly more complicated, globalized and pluralistic and, at the same time, devoid of universals, people living in it need more and more wisdom. Not the kind of wisdom that is found in modern technologies, but the kind that has a sapiential dimension and touches the foundations of life. People deprived of the meaning of their efforts will not be able to create societies that respect the principles of justice, peace, equality, freedom, human dignity, or the common good. Societies consisting of seemingly only happy Jobs, taking on more or less sophisticated masks of happy consumers, do not seem to have a future. On the one hand, we can see that the resources allowing the development of civilization are rapidly dwindling but, on the other hand, hopes for ever-increasing prosperity have already penetrated into the consciousness of people from every corner of the world. If we want to survive as human persons, mutually recognizing each other in our dignity, moral subjectivity, reasonableness, and autonomy, we must address the values that make our lives important and meaningful regardless of circumstances. This will not be possible unless there is an end to the trivialization and axiological banalization, as well as the decline of the virtues: sacrifice, generosity, humility, love, and valour.

Losing the perspective of spirituality, *sacrum* and eschatology, people will be forced to live in an atmosphere of existential anxiety that is intensified in relation to previous eras, though concealed at the same time. As Archbishop Józef Życiński aptly stated, reading the works of postmodern thinkers only leads to the conviction that the main component of the reality of our lives is the cold and mustiness. Thus, instead of creating any illusions that we live in a perfect world, it is better to accept the truth about the brutal nature of the world (Życiński, 2001). How the unfortunate Job can find himself in such a world, remains an open question? Is it even possible to exist in the affirmation of reality filled with the aforementioned “cold and mustiness”, especially while struggling with the misfortunes that affect us?

## Data Availability

All publicly available datasets referred to in this article are cited in reference list.
